# Working like a mule? The physiological toll of heavy loads on mules

**DOI:** 10.3389/fvets.2025.1725279

**Published:** 2025-12-02

**Authors:** Tamara Tadich, Javiera Calderón-Amor, Igor González, Bárbara Palma, Javiera Lagos

**Affiliations:** 1 Instituto de Ciencia Animal, Facultad de Ciencias Veterinarias, Universidad Austral de Chile, Valdivia, Chile; 2 Programa de Doctorado en Ciencias Veterinarias, Escuela de Graduados, Facultad de Ciencias Veterinarias, Universidad Austral de Chile, Valdivia, Chile; 3 Facultad de Ciencias de la Vida, Universidad de Viña del Mar, Viña del Mar, Chile; 4 Escuela de Medicina Veterinaria, Universidad de Chile, Santiago, Chile; 5 Programa de Doctorado en Ciencias Silvoagropecuarias y Veterinarias, Universidad de Chile, Santiago, Chile

**Keywords:** hybrid, workload, welfare, physiology, animal based indicators

## Abstract

**Background:**

Working mules are essential for human livelihoods, yet scientific evidence on their physiological limits during load-carrying tasks remains scarce.

**Objectives:**

To evaluate the physiological responses of mules to increasing loads during short-distance work.

**Study design:**

A crossover design. For this, 12 Chilean Army mules of three sizes (small, medium, large) were subjected to a 2-km walk carrying loads of 0, 80, 105, and 130 kg, equivalent to 20–42% of live body weight, under ambient temperatures of 32–39 °C. Blood and physiological parameters were measured before, immediately after, 10 min, and 2 h after work, with additional blood samples for biochemical parameters collected up to 105 h after exercise. Linear mixed models were applied to evaluate the effects of load, time, and body size.

**Results:**

The size of the mule did not have an effect on their physiological response to work. On the other hand, increasing load significantly elevated cortisol, lactate, and rectal temperature, indicating activation of both the hypothalamic–pituitary–adrenal axis and anaerobic metabolism. Enzymatic activities of γ-glutamyltransferase (GGT), lactate dehydrogenase (LDH), and alkaline phosphatase (SAP/ALP) also increased with heavier loads, suggesting greater muscular effort and mild tissue stress. Conversely, glutathione peroxidase (GPx) activity decreased as load increased, implying increased oxidative demand. Total protein and neutrophil-to-lymphocyte ratios rose with higher loads, reflecting systemic stress response and hemoconcentration. Heart rate and respiratory rate were not significantly affected, suggesting adequate cardiovascular adaptation. Most parameters returned to baseline within 2 h post-exercise, demonstrating effective short-term recovery.

**Conclusion:**

These findings confirm that short-distance work carrying heavy loads, in particular over 105 kg, does activate a physiological stress response in mules, though within adaptive limits. Loads of 105–130 kg triggered greater metabolic and enzymatic adjustments, indicating that while trained mules can cope with high loads over short distances, care should be taken when asking mules to work for longer distances or heavier loads since it can negatively affect their welfare. Establishing evidence-based workload thresholds is essential for balancing performance efficiency and welfare in working mules. Future studies should evaluate long-term work under field conditions, incorporating other welfare indicators such as behavior for a more comprehensive welfare assessment.

## 1 Introduction

Working equids are considered as an essential component of livelihood, and can be considered part of the natural, physical, social and financial capital of people (1) increasing resilience capacity of communities (2). The social and economic contribution of equids to livelihoods can be direct (provision of transportation services) or indirect (plowing the land for obtaining agricultural products), this is why underestimating their impact could negatively affect society (3).

Equids, and particularly mules, also remain being used as key elements for military purposes (4), playing a major role as animals of burden, messengers, protectors (5), and as an essential means of transport during natural disasters, when the army needs to access remote areas in order to rescue people or provide supplies. Mules have played a key role in transporting armaments, soldiers and their supplies (6), and become even more relevant in countries such as Chile with a long mountainous frontier, which can be of difficult access for mechanized vehicles and for horses.

Mules have been considered to be superior than horses for some types of work, because they are thought to have a higher endurance capacity, better quality of hooves, less feed requirements, and higher longevity/working life span (7–9). In mountainous areas they have a more secure step and a temperament that makes them an excellent pack animal compared with horses (7). Studies in different countries have shown a predilection for mules over donkeys and horses, but mules are usually less available and thus considered more valuable (8, 10). Nevertheless, little is known about their physiology and adaptation to work, and although they might not be displaying many signs of suffering, their welfare state could be compromised during some work activities.

Animal welfare is a multidimensional concept, the welfare of an individual has been defined by Broom (11) as “its state as regards its attempts to cope with its environment,” with an individual being able to cope with little efforts and expenditure of resources (energy) or it may fail to cope at all, in which case its welfare is poor. The term coping refers to the physiological and behavioral efforts (adjustments) of an animal to master a situation (12, 13), which can be assessed through animal-based measures of animal welfare, such as blood parameters. Army mules need to be able to cope with different types of work intensity, for example working with different loads, climate conditions and on difficult terrains. Studies assessing the welfare of working mules usually incorporate some animal-based measures as presence of wounds, inflammation processes, body condition scores and negative behaviors (14–17, 35). Nevertheless, there is a little amount of literature assessing their physiological response to work (18) and to our knowledge none with a controlled design.

Throughout exercise and training, the animal’s body should make certain physiological adjustments, adaptations or responses in a short term. If the animal is well adapted to its work, it has to perform it should be able to recover to baseline levels within a given time period. Mules from the army require training in order to adapt to work and avoid poor welfare. This adaptation can be assessed through their physiological adjustments (19), in order to estimate the impact of work and determine whether individuals are able to cope.

The factors that could directly affect the welfare of mules during work include endogenous and exogenous stressors (19). Among endogenous factors are those constraints induced by exercise, including physiological adjustments such as changes in blood composition. Exogenous stressors include the use of inefficient implements, overwork in terms of excessive load or long working hours, climatic conditions and inadequate interaction with the person leading the mule which may result in carelessness (7). Although equids are in general unique athletes, many of these stressors would not occur under natural conditions, and are mainly the result of overwork, poor training, and poor management.

During exercise, rapid changes occur in some blood parameters, most of them associated to the stress response and activation of the sympatho-adrenmedullar system (SAS) and the hypothalamic–pituitary–adrenal (HPA) axis. Red blood cells volume should be expected to increase, due to splenic contraction as a result of SAS activity (20). Cathecolamines, which promote glycogen breakdown in the muscle and liver and inhibition of insulin release, will also promote an increase of blood glucose concentration, increasing substrate availability for the working muscle (20). Glucocorticoids have been widely used as a stress and welfare indicator (21). The activation of the neuroendocrine stress response results in increases of serum cortisol concentrations that appear to better reflect duration of workload rather than intensity (20, 43), which together with changes in the neutrophil:lymphocyte (N: L) ratio seems to provide a reliable indicator of welfare (22). These parameters have been previously used to assess the effect of work in urban draft horses (23) finding significant changes after work, but with individuals being able to return to basal concentrations within 10 min afterwards. These parameters have not been assessed for packed work in mules.

Changes occurring during exercise can also lead to muscle cell fatigue and damage. Hyperkaliemia is expected as a result of loss by muscle cells during muscular contraction. Hyperkaliemia induces muscular fatigue by decreasing the muscular cell membrane potential causing conduction disturbance (19). Hyperlactacidemia can also be expected once the intensity of exercise reaches a certain level and energy starts to be provided by anaerobic metabolism (19, 43). This muscular acidemia is also responsible for muscular fatigue due to disturbance in mitochondrial function, impairment of glycolysis and decline in muscle ATP concentration. Blood lactate has shown to have a significant correlation with plasma ACTH, noradrenalin, and adrenalin response during exercise, which are sensitive indicators of exercise intensity (20, 24). When exercise is prolonged under this scenario (overwork) the result can be muscle strain leading to muscle damage and myopathy (25), particularly when individuals have not been trained properly. Muscular damage can result in inflammation resulting from high intensity work, particularly when carrying a load by means of implements that are not correctly positioned on the mules back, causing lesions. An increase in blood concentrations of enzymes that can be related to muscular damage can also be assessed such as creatinphosphokinase (CK), lactate deshydrogenase (LDH) and aspartate aminotransferase (AST). These markers have been previously used to assess the impact of rodeo exercise in Chilean horses and the effects of work in urban draft horses, finding significant increases (23, 26).

Hyperthermia can also be used to assess how the animal is coping with exercise. The muscular contraction that occurs during exercise will necessitate energy, which is provided by ATP degradation. Because conversion from stored chemical energy into mechanical energy is relatively inefficient, approximately 80% of energy will be lost as heat (19). Increased heart rate also occurs during work since the sympathoadrenal axis is activated in order to enhance oxygen delivery by stimulating the heart and increasing cardiac output up to 10-fold (20). For example, heart rate in horses can vary from 25–30 bpm to 220–240 bpm during maximal exercise, this would be the case of a race horse (19). The respiratory rate increases almost instantaneously in equids, their oxygen consumption and ventilation can increase by factors of more than 60 and 30, respectively, (19).

The type and extent of changes occurring during exercise will influence the time required for the animal to recover afterwards. For example, cathecolamine’s plasmatic half-life is of less than 30 s, and within a few minutes’ concentrations return to baselines (27), while recovery of glycogen stores is slower and can take up to 72 h to reach pre exercise concentrations. In cases where muscle damage occurs, soreness and stiffness may be detectable immediately after exercise or 1–3 days after. On the other hand, physiological changes associated to heart and respiratory rate, body temperature, pulse and oxygen saturation should recover pre exercise values between 10 min and 2 h afterwards (23, 28). These physiological parameters have been previously used as a measure of work intensity on Zanskar ponies carrying different loads (28). These differences in time should be taken into account in the design of the study in order to not underestimate or overestimate the physiological adjustments.

This is why the aim of this study was to assess the effect of three different loads on physiological indicators of the coping capacity of mules during a short distance work.

## 2 Materials and methods

This study was approved by the Animal Care and Use Committee of the Universidad de Chile, certificate N°19263-VET-UCH.

Animals: Twelve mules between 8 and 13 years old of three different sizes were selected (four small mules with an average height to the withers of 132cm and an average live weight of 325kg, four medium mules with and average height to the withers of 140cm and an average live weight of 363kg, and four large mules with an average height to the withers of 160cm and an average live weight of 383kg). The sizes were selected according to a previous morphological study on Chilean Army mules (29). They were crossbred mares with Boudet du Poitou or crossbred donkeys. Mules are bred by the Army at their breeding farms and at the age of 4 years they are destined to the units to start work. All mules belong to Detachment No. 3 “Yungay,” located in the city of Los Andes. During the trial, the mules were maintained on their routine diet, which consisted of 7 kg of alfalfa hay daily and water *ad libitum*.

Load Carrying Tests: During the end of spring (November and December), 12 mules were evaluated while traveling 2 km carrying loads of three different weights (L1: 80 kg, L2: 105 kg, and L3: 130 kg). The weight of each treatment includes the weight of the harnessing system plus bags of gravel until the total weight is reached. The trips were made at 10–12 day intervals (washout period), with four mules per weight assigned to each trip. These mules were randomly rotated on subsequent trips, using a crossover trial design. As a control, one trip without carrying weight (control treatment, L0) was also performed. For all trips mules were hand-led by a military, this means that each mule has a soldier who leads from the ground. This is the routine way in which packed mules are worked at the Army, meaning mules cannot choose their own speed.

Mules are usually housed in group (approximately 80 mules) in a paddock and hay is offered from the ground, while those that are being worked are housed individually for the days being exercised and they receive their hay in a trough. At the end of summer all mules are sent for a resting period to a larger field where they graze for approximately 45 days before returning to their routine.

The calculation of the transported weights was based on the weight of the packsaddle and harness normally used by Army mules (average 23 kg). The weight of the transported packs was then calculated for each treatment, using gravel as the load, subtracting the weight of the packsaddle. The 80 and 105 kg treatments carried two packs of equal weight (one tied to each side of the packsaddle), and the 130 kg treatment added a third pack, tied to the dorsal area of the packsaddle.

Before each experiment, a clinical examination was performed to determine the health status, ruling out mules with signs of illness. The examination consisted in ruling out signs of lameness, pain, lesions, fever and abnormalities associated to heart and respiratory rate. Seven blood sampling times were established: T0: before starting work; T1: immediately after work; T2: 10 min after completing the 2km walk; T3: 2 h after work; T4: 24 h after work; T5: 72 h after work and T6: 105 h after work.

Heart rate (HR), respiratory rate (RR), rectal temperature (RT), cortisol, lactate, and glucose were assessed only at T0, T1, T2, and T3. Lactate and glucose were measured in the field using test strips and a Roche® Accutrend Plus meter and a Uright® Plus 1 meter, respectively. HR was assessed using a stethoscope, RR was assessed by observing chest and abdominal movement, and rectal temperature was measured using a digital thermometer.

Blood samples: Prior to the experiments, all mules underwent jugular vein catheterization to facilitate sample collection. At each sampling time, 15 mL of blood were obtained and divided into three tubes: one with EDTA for hematology, one with heparin for the glutathione peroxidase (GPx) enzyme, and one without additives for the biochemical profile and cortisol concentration. The variables evaluated were the following: Hematological variables (Agglomerated red blood cell volume, N: L ratio, and total proteins); Biochemical variables [Glucose, lactate, potassium, creatine kinase (CK), lactate dehydrogenase (LDH), aspartate aminotransferase (AST), gamma-glutamyltransferase (GGT), Alkaline Phosphatase (SAP/ALP) and glutathione peroxidase (GPx)]; Hormones [Cortisol (CORT)]. All blood analyses were done as in Lagos and Tadich (30).

Other variables registered were ambient temperature, humidity, altitude and slope using a GPS device (Garmin® forerunner 645) and an anemometer (Benetech® GM816).

All statistical analyses were conducted using R software version 4.1.0 (R Development Core Team, 2021®). All blood variables were corrected according to their baseline value and this difference was used in the statistical analyses.

We employed linear mixed models via the lme4 package (44), utilizing the lmer function to explore the associations between explanatory factors and blood parameters. The independent variables considered included Time (T0 to T6), Load (0 kg, 80 kg, 105 kg, 130 kg), and Body Size (Small, Medium, Large), alongside interactions between Time and Load. Each mule was treated as a random effect to account for repeated measures. For physiological parameters heart rate, respiratory rate, temperature, glucose, lactate, and cortisol, analyses were limited to the first three sampling times.

To evaluate the effects of the explanatory variables, we used the Type II Wald chi-square tests implemented in the car package (31). Significant effects were further examined using Tukey’s *post-hoc* adjustments with the emmeans package (32). Preliminary checks for the normality of residuals and homogeneity of variances were performed visually in all models.

The models were specified as follows, with an example for the response variable cortisol:

Cortisol∼Time+Load+Time×Load+Size+(1∣Mule) .

## 3 Results

The 2-km walk was conducted in November and December of 2019, with temperatures ranging from 32 to 39 °C, an average relative humidity of 24%, and an altitude of 856 to 878 meters above sea level. The walk was conducted at an average speed of 4.6 km/h (2.8 mph) and a pace of 12.4 min/km (1.1 mph). The proportion of weight carried was between 20 and 42% of the mules’ live weight (LW), depending on the size of the mule (small, medium and large) and the load carried (80, 105 or 130kg).

Descriptive statistics for all blood parameters categorized by load and sampling time are presented in Table 1. Table 2 summarizes short-term blood responses associated with Glucose, Lactate and Cortisol according to load and sampling time, while descriptive statistics for physiological parameters (heart rate, respiratory rate and rectal temperature) are shown in Table 3 according to load and sampling time. Detailed results from the linear mixed models and *post-hoc* analyses are provided in Supplementary Annex 1, 2.

**Table 1:** Descriptive statistics (mean ± standard deviation) for each hematological variable across different load conditions and sampling times.

Variable	Treatment	Sampling time						
		T_0_	T_1_	T_2_	T_3_	T_4_	T_5_	T_6_
Potassium (mmol/L)	Control	4.73 ± 0.2	4.84 ± 9.1	4.75 ± 0.3	4.79 ± 0.4	4.66 ± 0.6	5.05 ± 0.3	4.79 ± 0.4
	80kg	4.94 ± 0.3	4.94 ± 0.4	4.8 ± 0.6	4.65 ± 0.4	4.79 ± 0.5	4.45 ± 0.9	4.48 ± 0.6
	105kg	4.21 ± 0.32	4.25 ± 0.4	4.12 ± 0.5	3.87 ± 0.6	4.12 ± 0.3	3.97 ± 1.0	4.65 ± 6.3
	130kg	4.07 ± 0.2	3.98 ± 0.3	3.93 ± 0.2	3.82 ± 0.2	4.08 ± 0.2	4.18 ± 0.4	4.41 ± 0.3
Albumin (g/L)	Control	32.9 ± 1.93	31.8 ± 1.6	31.5 ± 1.8	31.5 ± 1.6	31.3 ± 1.9	31.1 ± 3.53	32.0 ± 2.0
	80kg	33.7 ± 3.3	32.8 ± 10.1	35.6 ± 3.6	34.5 ± 4.4	33.9 ± 4.2	33.2 ± 3.4	33.2 ± 2.4
	105kg	30.8 ± 3.7	31.9 ± 10.0	31.4 ± 4.8	29.8 ± 3.1	32.3 ± 2.6	32.1 ± 3.2	33.4 ± 4.2
	130kg	31.3 ± 2.5	33.4 ± 2.3	33.3 ± 2.7	31.6 ± 4.2	30.1 ± 3.6	29.6 ± 3.9	32.6 ± 2.9
CK (IU/L)	Control	238.3 ± 54.3	233.8 ± 87.6	233.3 ± 64.1	183.2 ± 41.8	186.3 ± 37.6	171.7 ± 44.5	174.3 ± 108.2
	80kg	204.7 ± 50.7	216.3 ± 43.2	203.3 ± 43.4	192.6 ± 42.7	145.3 ± 45.5	140.5 ± 72.8	193.4 ± 102.7
	105kg	238.8 ± 141.0	253.2 ± 108.3	223.5 ± 101.2	188.5 ± 98.6	206.3 ± 96.4	220.1 ± 108.4	366.6 ± 492.6
	130kg	287.9 ± 152.3	332.5 ± 207.3	307.3 ± 144.2	222.2 ± 126.2	200.1 ± 80.1	247.8 ± 111.3	182.3 ± 60.1
LDH (IU/L)	Control	792.08 ± 88.5	823.3 ± 145.9	808.8 ± 143.3	654.8 ± 120.8	587.7 ± 124.9	482.3 ± 104.1	465.4 ± 110.1
	80kg	640.9 ± 238.1	677.2 ± 207.1	632.7 ± 211.9	533.4 ± 290.2	420.9 ± 109.0	347.1 ± 134.8	408.9 ± 131.3
	105kg	540.2 ± 186.5	594.4 ± 191.1	453.0 ± 191.7	427.3 ± 96.7	513.1 ± 164.8	526.9 ± 131.4	574.8 ± 154.3
	130kg	781.9 ± 271.1	834.6 ± 196.9	805.3 ± 235.4	627.6 ± 265.3	601.5 ± 192.7	719.8 ± 212.3	575.0 ± 172.5
ALP/SAP (IU/L)	Control	242 ± 41.5	235.6 ± 46.5	234.2 ± 44.4	228.5 ± 43.6	229.1 ± 42.2	222.2 ± 46.3	232.2 ± 73.0
	80kg	189.7 ± 48.2	215.5 ± 54.4	191.7 ± 53.6	195.9 ± 68.9	194.5 ± 45.7	189.0 ± 57.9	204.7 ± 74.9
	105kg	267.8 ± 57.6	258.4 ± 90.6	275.7 ± 59.3	279.8 ± 59.6	287.8 ± 63.1	305.8 ± 53.3	308.7 ± 64.4
	130kg	328.8 ± 73.7	348.7 ± 74.0	350.9 ± 79.1	343 ± 71.4	323.1 ± 69.8	337.3 ± 73.4	358.3 ± 94.5
AST (IU/L)	Control	296.5 ± 26.8	303.7 ± 52.5	295.1 ± 28.3	287.8 ± 36.6	292.6 ± 23.3	304.6 ± 30.9	321.8 ± 50.8
	80kg	282.7 ± 49.2	304.1 ± 50.1	298.0 ± 49.5	306.0 ± 49.7	297.7 ± 46.7	280.0 ± 68.8	293.3 ± 55.5
	105kg	233.6 ± 32.0	231.1 ± 76.1	248.8 ± 61.0	241.3 ± 46.1	240.8 ± 42.1	233.1 ± 62.8	235.1 ± 42.3
	130kg	226.4 ± 38.1	212.2 ± 58.6	239.7 ± 49.0	243.8 ± 61.6	243.2 ± 44.9	234.0 ± 38.3	241.8 ± 30.5
GGT (IU/L)	Control	38.5 ± 10.8	34.1 ± 12.02	34.6 ± 10.1	27.5 ± 8.4	28.0 ± 7.1	26.9 ± 19.8	22.8 ± 10.4
	80kg	35.5 ± 9.03	34.0 ± 8.4	30.8 ± 7.4	25.8 ± 7.2	21.1 ± 5.9	23.9 ± 20.9	22.6 ± 9.7
	105kg	31.8 ± 7.4	32.6 ± 10.5	27.3 ± 5.3	25.6 ± 5.5	26.5 ± 7.4	29.3 ± 8.9	30.4 ± 12.8
	130kg	31.5 ± 13.9	34.8 ± 12.2	33.3 ± 11.9	28.2 ± 10.6	27.3 ± 9.9	33.1 ± 11.8	31.5 ± 12.2
GPx (IU/g Hb)	Control	107.2 ± 35.7	104.2 ± 39.4	110.5 ± 53.8	111.4 ± 48.4	132.8 ± 35.5	143.2 ± 27.1	137.7 ± 30.2
	80kg	179.4 ± 21.2	179.6 ± 29.8	165.6 ± 26.2	172.9 ± 15.8	171.9 ± 21.8	163.8 ± 21.6	170.1 ± 27.9
	105kg	211.7 ± 80.6	189.0 ± 65.7	189.1 ± 61.3	194.7 ± 67.8	185.4 ± 64.2	195.3 ± 54.9	190.0 ± 61.3
	130kg	134.0 ± 25.9	130.9 ± 16.9	136.8 ± 21.3	134.5 ± 28.9	120.5 ± 19.1	127.1 ± 29.0	120.0 ± 49.2
ARBCV (%)	Control	32.4 ± 3.9	32.2 ± 2.8	32.6 ± 2.2	31.4 ± 3.5	31.8 ± 2.9	35.2 ± 6.3	31.7 ± 4.0
	80kg	31.9 ± 2.9	34.6 ± 4.5	33.6 ± 2.9	31.6 ± 2.8	34.2 ± 4.1	32.6 ± 4.3	33.6 ± 3.7
	105kg	32.3 ± 3.4	32.9 ± 2.9	33.0 ± 2.1	31.8 ± 2.4	32.3 ± 3.1	32.4 ± 2.7	34.5 ± 5.6
	130kg	32.9 ± 4.4	34.4 ± 3.2	34.1 ± 3.4	30.2 ± 2.9	31.2 ± 1.9	31.8 ± 4.2	33.4 ± 3.6
Neutrophil Limphocyte ratio (N: L)	Control	2.2 ± 0.8	1.8 ± 0.9	2.3 ± 1.5	1.8 ± 0.8	1.5 ± 0.5	2.3 ± 1.4	1.9 ± 0.7
	80kg	2.0 ± 0.9	2.1 ± 2.1	2.0 ± 0.9	2.2 ± 1.1	1.8 ± 0.6	1.6 ± 1.2	1.6 ± 0.9
	105kg	1.6 ± 0.6	2.0 ± 0.6	2.3 ± 0.9	2.6 ± 0.9	2.6 ± 1.3	2.1 ± 1.0	1.7 ± 0.7
	130kg	2.5 ± 1.5	2.2 ± 1.1	2.2 ± 1.3	2.4 ± 1.2	2.3 ± 1.2	1.6 ± 1.0	1.5 ± 0.5
Total proteins (g/L)	Control	72.5 ± 2.9	72.2 ± 4.9	71.7 ± 5.4	72.0 ± 4.9	71.3 ± 5.2	70.2 ± 8.2	72.4 ± 5.8
	80kg	72.9 ± 8.3	77.5 ± 5.7	77.2 ± 8.2	75.7 ± 5.1	72.3 ± 4.6	70.9 ± 8.7	71.6 ± 10.7
	105kg	73.3 ± 7.1	77.3 ± 8.0	75.3 ± 3.4	73.8 ± 4.7	76.8 ± 5.3	79.0 ± 5.0	78.6 ± 12.5
	130kg	79.7 ± 9.5	83.1 ± 9.6	81.8 ± 8.4	82.0 ± 5.8	78.0 ± 6.5	78.4 ± 7.2	82.8 ± 11.2

**Table 2:** Descriptive statistics (mean ± standard deviation) for each short term blood variables (Glucose, Lactate and Cortisol) across different load conditions and sampling times.

Variable	Treatment	Sampling times			
		T_0_	T_1_	T_2_	T_3_
Glucose (mg/dL)	Control	57.8 ± 9.2	54.5 ± 11.6	57.7 ± 5.5	65.3 ± 4.5
	80kg	70.6 ± 11.0	64.5 ± 4.9	66.3 ± 4.7	71.8 ± 4.5
	105kg	69.2 ± 13.8	66.3 ± 5.4	64.3 ± 8.4	72.4 ± 7.0
	130kg	64.4 ± 6.9	66.6 ± 5.0	63.9 ± 6.0	71.0 ± 8.1
Lactate (mmol/L)	Control	2.2 ± 0.8	1.6 ± 0.4	1.7 ± 0.6	2.1 ± 0.8
	80kg	1.9 ± 0.6	2.0 ± 0.6	2.2 ± 0.6	2.0 ± 0.6
	105kg	1.9 ± 0.6	1.7 ± 0.9	2.1 ± 0.8	2.1 ± 0.7
	130kg	1.9 ± 0.4	2.2 ± 1.2	2.1 ± 0.9	2.5 ± 0.6
Cortisol (nmol/L)	Control	62.8 ± 34.5	81.1 ± 25.7	77.0 ± 29.0	68.5 ± 36.4
	80kg	83.0 ± 28.1	114.7 ± 40.6	114.1 ± 39.7	88.3 ± 31.0
	105kg	77.4 ± 26.2	137.8 ± 55.4	136.5 ± 53.7	94.4 ± 39.0
	130kg	71.8 ± 21.4	137.5 ± 34.5	135.6 ± 34.7	83.6 ± 19.4

**Table 3:** Descriptive statistics (mean ± standard deviation) for each physiological variable (Heart rate, Respiratory rate and Rectal temperature) across different load conditions and sampling times.

Variable	Treatment	Sampling time			
		T_0_	T_1_	T_2_	T_3_
Heart rate (HR) (bpm)	Control	40.3 ± 6.9	53.2 ± 18.8	44.0 ± 5.1	40.8 ± 6.5
	80kg	36.9 ± 11.3	57.5 ± 18.4	46.0 ± 13.8	36.9 ± 8.5
	105kg	40.2 ± 9.7	65.0 ± 18.5	48.8 ± 9.9	39.8 ± 5.6
	130kg	42.3 ± 4.9	65.6 ± 11.9	51.8 ± 8.0	40.3 ± 5.3
Respiratory rate (RR) (cycles per min)	Control	22.7 ± 7.5	41.7 ± 11.9	31.2 ± 10.8	19.3 ± 4.8
	80kg	18.8 ± 9.8	30.7 ± 15.0	25.3 ± 8.3	19.6 ± 4.6
	105kg	17.2 ± 5.5	31.5 ± 13.7	31.5 ± 13.2	19.3 ± 4.2
	130kg	17.2 ± 7.3	37.3 ± 10.5	30.0 ± 7.9	21.2 ± 5.3
Rectal temperature (C°)	Control	37.1 ± 0.5	37.6 ± 0.4	37.7 ± 0.4	37.2 ± 0.3
	80kg	36.0 ± 0.8	37.1 ± 0.6	37.0 ± 0.6	36.7 ± 0.5
	105kg	36.1 ± 0.7	36.7 ± 0.6	36.7 ± 0.6	36.6 ± 0.7
	130kg	36.4 ± 0.7	37.1 ± 0.5	37.0 ± 0.6	36.6 ± 0.7

### 3.1 Changes in biochemical parameters

For Potassium, the statistical analysis showed a significant interaction between time and load (χ^2^ = 33.6, *p* = 0.003). At T5, increasing the load from 0 kg to 105 kg significantly reduced potassium concentrations (*p* = 0.001), while an increase was observed from 105 kg to 130 kg (*p* = 0.01). At T6, increasing the load from 80 to 105 kg (*p* = 0.007) and to 130 kg (*p* = 0.009) also significantly elevated potassium levels. The main effect of load had a significant effect, reducing potassium levels from 0 kg to 80 kg (*p* = 0.001) and from 0 kg to 105 kg (*p* = 0.019). Body size had no significant effect.

When looking at Total proteins the main effect of load was significant (χ^2^ = 22.75, *p* < 0.0001), with total protein concentrations rising with increases in load from 0 kg to 105 kg (*p* < 0.0001) and 130 kg (*p* = 0.04). Neither the interaction between time and load nor body size affected protein levels.

For enzymes, GGT concentrations were significantly influenced by load (χ^2^ = 83.79, *p* < 0.0001). Increases in load from 0 kg to 105 kg (*p* < 0.0001) and from 0 kg to 130 kg (*p* < 0.0001) corresponded with higher concentrations of GGT. There were no significant effects from the interaction between time and load or from body size. CK showed a significant main effect of load (χ^2^ = 7.94, *p* = 0.04), although *post-hoc* tests indicated no significant differences. The interaction between time and load, and body size showed no effect. SAP concentrations also showed a significant response to load changes (χ^2^ = 23.04, *p* < 0.0001). Higher loads consistently resulted in elevated SAP concentration across the increments from 0 kg to 80 kg (*p* = 0.006), 105 kg (*p* = 0.0003), and 130 kg (*p* = 0.0004). The interactions between time and load, as well as body size, showed no effect. LDH concentrations showed a significant interaction between time and load (χ^2^ = 46.84, *p* < 0.0001). *Post-hoc* tests indicated that, at T5, an increase in load from 0 kg to 105 kg (*p* < 0.0001) and from 0Kg to 130Kg (*p* = 0.007) led to a significant increase in LDH. Additionally, at T6, elevating the load from 0kg to 105 kg (*p* < 0.0001) also resulted in a significant increase in LDH concentration. The main effect of load was also significant (χ^2^ = 31.33, *p* < 0.0001). At 105 Kg LDH showed the highest concentration compared to 0Kg (*p* < 0.0001), 80Kg (*p* < 0.0001), and 130Kg (*p* = 0.02). Body size showed no effect. GPx concentrations showed a significant main effect of load (χ^2^ = 39.25, *p* < 0.0001); GPx decreased when load increased from 0Kg to 80Kg (*p* = 0.002), 0Kg to 105Kg (*p* < 0.0001), and from 0Kg to 130Kg (*p* = 0.005). The interaction between time and load, and body size showed no effect. Finally, AST showed no significant association with any of the variables.

For variables associated to the hematocrit, the neutrophil:lymphocyte ratio (N: L) showed a significant main effect of load (χ^2^ = 28.38, *p* < 0.0001); N: L increased when load increased from 0Kg to 105Kg (*p* = 0.0008) and from 80 kg to 105 kg (*p* = 0.008), but decreased again at 130 kg (*p* < 0.0001). The interaction between time and load, and body size showed no effect. In addition, the Aggregated red blood cell volume (ARBCV) showed a significant response to changes in load (χ^2^ = 8.45, *p* = 0.003). The ARBCV diminished when load increased from 80 kg to 130 kg (*p* = 0.02). The interaction between time and load, and body size had no significant impact.

### 3.2 Short-term responses

For Cortisol, the main effect of load was significant (χ^2^ = 40.85, *p* = 0.003); Cortisol concentration increased when load increased from 0Kg to 80Kg (*p* = 0.04), from 0Kg to 105Kg (*p* < 0.0001), and from 0Kg to 130Kg (*p* < 0.0001). The interaction between time and load, and body size showed no effect. For Glucose, the main effect of load was significant (χ^2^ = 9.26, *p* = 0.02); but *post-hoc* test showed no significant differences. The interaction between time and load, and body size showed no effect. Finally, Lactate showed a significant main effect of load (χ^2^ = 16.81, *p* = 0.0007); lactate levels increased when load increased from 0Kg to 80Kg (*p* = 0.05), from 0Kg to 105Kg (*p* = 0.007), and from 0Kg to 130Kg (*p* = 0.002). The interaction between time and load, and body size showed no effect.

### 3.3 Physiological variables

There was no significant effect of load or time on Heart rate (HR) and Respiratory rate (RR). For Temperature, the main effect of load was significant (χ^2^ = 12.89, *p* = 0.004) with a significant increase observed as load increased from 0 kg to 80 kg (*p* = 0.007), but then showed a slight decrease at 130 kg (*p* = 0.02). The interaction between time and load, and body size showed no effect.

## 4 Discussion

The results obtained in this study confirm that an increase in the load carried by mules causes physiological and biochemical changes consistent with greater activation of stress response mechanisms and an increase in the metabolic demand associated with work. In this study, a short distance work of 2km was enough to challenge the mules coping capacity, in particular with loads of 130kg.

In general terms, for all loads, the speed at which mules performed the work did not vary from an average of 4.6 km/h. Although equines adjust their speed in order to optimize their metabolic rate (33), in this study they were hand-led by a military, thus they were not able to choose their own speed. Nevertheless, this speed was higher than that reported for working donkeys by Legha et al. (34). This could be also related to the size of donkeys (110 to 142kg), the fact that they were carrying heavier loads (between 40 and 66% of their LBW) and for a longer distance (up to 9km).

The increase in cortisol and lactate observed with increasing load demonstrates the activation of both the hypothalamic–pituitary–adrenal (HPA) axis and anaerobic metabolism, mechanisms that enable them to cope with situations of intense physical exertion (20, 43). In this sense, the increase in cortisol observed even in short-term work indicates that the load represents a significant stressor, albeit within a physiological range compatible with an adaptive response. Two hours after work (T3), cortisol and lactate already showed concentrations similar to baseline before work. Similar results have been described in urban draft horses subjected to moderate work (23), suggesting that mules exhibit response patterns comparable to those of other equine species. It is important to emphasize that this was a short distance work, and already a significant increase was observed at higher loads. Future studies should be conducted under real conditions, when mules travel for days up the Andes mountains.

Increases in enzymes such as GGT, LDH, SAP/ALP, and CK at higher loads reinforce the interpretation that overload work involves some degree of additional muscular effort and possible mild tissue damage, consistent with previous reports by Snow and Valberg (25) and Art and Lekeux (19). Nevertheless, with the exemption of LDH, all increases were still within normal intervals for mules (30). The presence of a mild inflammation for higher loads is in line with Lagos et al. (35) who described an increase in mule’s back temperature with loads of 105 and 130Kg and of inflammation markers such as Serum Amiloid A (SAA) at 72 and 120 h after work. However, the magnitude of these changes and the absence of clinical signs of fatigue or persistent alterations suggest that the mules were able to regain homeostasis in the hours following work. This is consistent with the idea that well-trained and adapted animals can restore their baseline parameters within a limited period (23, 28).

In the case of GPx, this antioxidant enzyme showed a significant decrease when increasing load. According to a recent meta-analysis by Xie et al. (36) exercise has a significant effect on increasing GPx activity, but with an important heterogeneity between studies, since factors such as gender, season and exercise intensity can have an effect. For example, in Shetland ponies, Kurhaluk et al. (37) found a decrease in this enzyme after exercise, while Vergara and Tadich (23) found no differences after work in urban draft horses used for tourism. Nevertheless, it is important to highlight that average GPx concentration in most sampling times were closer to the lower normal interval limit reported for Chilean mules (30), thus selenium and vitamin E supplementation could be something to consider for working mules, in particular in Chile were forage is poor in this mineral due to volcanic soils (38).

The significant increase in total protein and the neutrophil:lymphocyte ratio with higher loads could reflect both hemoconcentration due to fluid loss during exercise and the influence of glucocorticoids on leukocyte redistribution (22). These results complement the endocrine interpretation of the stress response, demonstrating coordinated systemic activation. We should also consider that in this study potassium concentrations increased significantly when load was increased from 105 to 130kg, but decreased at lower loads. Increases in plasma potassium have been associated to intense exercise when muscle contraction generates an efflux from the muscle cell (45), nevertheless potassium concentration in the present study was always within normal limits (30). On the other hand, a decrease in potassium can be associated to dehydration (39), and although donkeys present less sweating than horses and are considered more tolerant to high temperatures (40), dehydration has not been studied in depth in mules.

After the 2km walk, changes were found in some physiological variables, indicating adjustments in response to exercise (34, 41). For example, HR, RR and rectal temperature are some of the main indicators of the level of effort resulting from exercise in horses (46) and their increase varies depending on the intensity of the exercise performed (42). This is why the progression in the recovery of pre-exercise concentrations is an indicator of adaptation to the physical effort performed (46, 47, 23), so it is expected that an animal adapted to exercise will recover its basal values in a short period of time of approximately 10 min (T2 of this study). Although HR and RR did increase after work (T1), these increases were not significant for any of the loads. On the other hand, rectal temperature increased with increasing load, consistent with exercise-associated metabolic heat production (19). However, the stability of heart and respiratory rates suggests that the duration and intensity of the work were not sufficient to compromise the mules’ thermoregulatory capacity or cardiovascular efficiency. This finding could indicate a good level of training and adaptation to the environmental conditions of the area (32–39 °C, 24% RH), which is relevant considering the operational role these animals play in mountain environments and extreme temperatures.

The results confirm that body size did not have a significant effect on physiological indicators, which is consistent with previous observations indicating that mules, regardless of size, exhibit high efficiency in energy conversion during work (7, 8). This is relevant, since when working in mountainous terrain a large mule may not be optimal. For work, and an animal with good temperament, proper training and with its center of gravity closer to the ground should be preferred. This, together with the fact that military prefer working with medium-sized mules (29), should be considered when breeding them.

Overall, the results support the need to define safe work limits based on physiological evidence, especially in working animals, where efficiency and welfare must be balanced. Although the mules demonstrated adequate capacity to cope with high loads over short distances, the repetition of these conditions or their prolonged duration could lead to cumulative fatigue and the risk of muscular or metabolic injuries. The tendency toward greater variations in some parameters with the loads of 105 kg and 130kg suggests that the current 105kg work threshold might be correct for short period works, but for higher intensity works high loads will translate into a greater physiological demand. Therefore, future studies should evaluate the effects of prolonged hours of work and distances, different slopes, and longer recovery periods, while also integrating behavioral and performance indicators for a more comprehensive assessment of the mules’ welfare.

## Data Availability

The original contributions presented in the study are included in the article/Supplementary material, further inquiries can be directed to the corresponding author.
