# Supplementary material for: Working like a mule? The physiological toll of heavy loads on mules
Source: Front Vet Sci. 2025 Dec 2;12:1725279. doi: 10.3389/fvets.2025.1725279 (PMC12706672; doi:10.3389/fvets.2025.1725279)
Supplement: Supplementary file 1 [file Table_1.docx]

Supplementary Material

**Annexe 1**. Effects of explanatory variables were computed using the ‘Anova’ function (‘car’ R package), running Type II Wald chisquare trials. All linear models were computed using the ‘lmer’ function, considering repeated observations as random factors (individual). All the models by blood variable (dependent variable) and the explanatory variables Time, Load, Size, and the interaction between Time and Load are shown. P values were considered significant when below 0.05.

| **Predictor** | **Chisq** | **Df** | **Pr.Chisq.** |
| --- | --- | --- | --- |
| **Potassium** |  |  |  |
| Time | 17.65 | 5 | **0.003** |
| Load | 19.94 | 3 | **0.0001** |
| Size | 0.69 | 2 | 0.70 |
| Time*Load | 33.68 | 15 | **0.003** |
| **Total proteins** |  |  |  |
| Time | 8.10 | 5 | 0.15 |
| Load | 22.75 | 3 | **<0.0001** |
| Size | 0.15 | 2 | 0.92 |
| Time*Load | 22.80 | 15 | 0.09 |
| **GGT** |  |  |  |
| Time | 33.81 | 5 | **<0.0001** |
| Load | 83.79 | 3 | **<0.0001** |
| Size | 0.65 | 2 | 0.71 |
| Time*Load | 24.23 | 15 | 0.06 |
| **CK** |  |  |  |
| Time | 15.12 | 5 | **0.009** |
| Load | 7.92 | 3 | **0.04** |
| Size | 0.06 | 2 | 0.96 |
| Time*Load | 23.29 | 15 | 0.07 |
| **SAP** |  |  |  |
| Time | 4.24 | 5 | 0.51 |
| Load | 23.04 | 3 | **<0.0001** |
| Size | 2.88 | 2 | 0.23 |
| Time*Load | 19.35 | 15 | 0.19 |
| **LDH** |  |  |  |
| Time | 56.73 | 5 | **<0.0001** |
| Load | 31.33 | 3 | **<0.0001** |
| Size | 0.63 | 2 | 0.72 |
| Time*Load | 45.84 | 15 | **<0.0001** |
| **GP** |  |  |  |
| Time | 1.63 | 5 | 0.89 |
| Load | 39.25 | 3 | **<0.0001** |
| Size | 4.88 | 2 | 0.08 |
| Time*Load | 15.68 | 15 | 0.40 |
| **AST** |  |  |  |
| Time | 2.74 | 5 | 0.73 |
| Load | 1.59 | 3 | 0.66 |
| Size | 1.94 | 2 | 0.37 |
| Time*Load | 17.09 | 15 | 0.31 |
| **NL** |  |  |  |
| Time | 5.91 | 5 | 0.31 |
| Load | 28.38 | 3 | **<0.0001** |
| Size | 0.35 | 2 | 0.83 |
| Time*Load | 9.03 | 15 | 0.87 |
| **VGA** |  |  |  |
| Time | 16.54 | 5 | **0.005** |
| Load | 8.45 | 3 | **0.03** |
| Size | 0.24 | 2 | 0.88 |
| Time*Load | 21.33 | 15 | 0.12 |
| **Cortisol** |  |  |  |
| Time | 51.20 | 2 | **<0.0001** |
| Load | 40.85 | 3 | **<0.0001** |
| Size | 3.40 | 2 | 0.18 |
| Time*Load | 11.17 | 6 | 0.08 |
| **Glucose** |  |  |  |
| Time | 21.60 | 2 | **<0.0001** |
| Load | 9.26 | 3 | **0.02** |
| Size | 4.34 | 2 | 0.11 |
| Time*Load | 2.66 | 6 | 0.85 |
| **Lactate** |  |  |  |
| Time | 3.61 | 2 | 0.16 |
| Load | 16.81 | 3 | **0.0007** |
| Size | 0.15 | 2 | 0.92 |
| Time*Load | 4.43 | 6 | 0.61 |
| **Heart rate** |  |  |  |
| Time | 64.44 | 2 | **<0.0001** |
| Load | 3.83 | 3 | 0.28 |
| Size | 0.41 | 2 | 0.81 |
| Time*Load | 4.57 | 6 | 0.59 |
| **Temperature** |  |  |  |
| Time | 12.51 | 2 | **0.001** |
| Load | 12.89 | 3 | **0.004** |
| Size | 2.26 | 2 | 0.32 |
| Time*Load | 2.40 | 6 | 0.87 |

**Annexe 2:** Post hoc tests showing significant interactions or single effects of explanatory variables (Load and Time*Load). Each model is indicated by blood parameter and all post hoc tests were computed using the ’lsmeans’ function with Tukey correction for multiple testing.

| **Load** | **Time** | **estimate** | **SE** | **df** | **t.ratio** | **p.value** |
| --- | --- | --- | --- | --- | --- | --- |
| **Potassium** |  |  |  |  |  |  |
| **Time*Load** |  |  |  |  |  |  |
| 0KG - 105KG | T1 | 0.06 | 0.17 | 244.01 | 0.36 | 0.98 |
| 0KG - 130KG | T1 | 0.13 | 0.17 | 244.01 | 0.73 | 0.88 |
| 0KG - 80KG | T1 | 0.15 | 0.17 | 244.47 | 0.85 | 0.83 |
| 105KG - 130KG | T1 | 0.06 | 0.17 | 244.01 | 0.38 | 0.98 |
| 105KG - 80KG | T1 | 0.09 | 0.17 | 244.47 | 0.50 | 0.96 |
| 130KG - 80KG | T1 | 0.02 | 0.17 | 244.47 | 0.14 | 1.00 |
| 0KG - 105KG | T2 | 0.17 | 0.17 | 244.01 | 1.02 | 0.74 |
| 0KG - 130KG | T2 | 0.12 | 0.17 | 244.01 | 0.71 | 0.89 |
| 0KG - 80KG | T2 | 0.11 | 0.17 | 244.47 | 0.62 | 0.93 |
| 105KG - 130KG | T2 | -0.05 | 0.17 | 244.01 | -0.31 | 0.99 |
| 105KG - 80KG | T2 | -0.07 | 0.17 | 244.47 | -0.38 | 0.98 |
| 130KG - 80KG | T2 | -0.01 | 0.17 | 244.47 | -0.08 | 1.00 |
| 0KG - 105KG | T3 | 0.46 | 0.17 | 244.36 | 2.61 | 0.05 |
| 0KG - 130KG | T3 | 0.28 | 0.17 | 244.01 | 1.65 | 0.35 |
| 0KG - 80KG | T3 | 0.38 | 0.17 | 244.47 | 2.17 | 0.13 |
| 105KG - 130KG | T3 | -0.17 | 0.17 | 244.36 | -1.00 | 0.75 |
| 105KG - 80KG | T3 | -0.08 | 0.18 | 244.87 | -0.43 | 0.97 |
| 130KG - 80KG | T3 | 0.10 | 0.17 | 244.47 | 0.56 | 0.94 |
| 0KG - 105KG | T4 | 0.07 | 0.17 | 244.01 | 0.41 | 0.98 |
| 0KG - 130KG | T4 | -0.11 | 0.17 | 244.01 | -0.66 | 0.91 |
| 0KG - 80KG | T4 | 0.07 | 0.17 | 244.47 | 0.37 | 0.98 |
| 105KG - 130KG | T4 | -0.18 | 0.17 | 244.01 | -1.07 | 0.71 |
| 105KG - 80KG | T4 | 0.00 | 0.17 | 244.47 | -0.03 | 1.00 |
| 130KG - 80KG | T4 | 0.18 | 0.17 | 244.47 | 1.01 | 0.74 |
| 0KG - 105KG | T5 | 0.73 | 0.17 | 244.01 | 4.29 | 0.001 |
| 0KG - 130KG | T5 | 0.18 | 0.17 | 244.01 | 1.06 | 0.72 |
| 0KG - 80KG | T5 | 0.63 | 0.18 | 244.84 | 3.49 | 0.002 |
| 105KG - 130KG | T5 | -0.55 | 0.17 | 244.01 | -3.23 | 0.01 |
| 105KG - 80KG | T5 | -0.11 | 0.18 | 244.84 | -0.59 | 0.94 |
| 130KG - 80KG | T5 | 0.45 | 0.18 | 244.84 | 2.49 | 0.06 |
| 0KG - 105KG | T6 | -0.26 | 0.17 | 244.01 | -1.51 | 0.43 |
| 0KG - 130KG | T6 | -0.31 | 0.17 | 244.01 | -1.81 | 0.27 |
| 0KG - 80KG | T6 | 0.36 | 0.18 | 244.84 | 2.03 | 0.18 |
| 105KG - 130KG | T6 | -0.05 | 0.17 | 244.01 | -0.30 | 0.99 |
| 105KG - 80KG | T6 | 0.62 | 0.18 | 244.84 | 3.47 | 0.007 |
| 130KG - 80KG | T6 | 0.67 | 0.18 | 244.84 | 3.76 | 0.009 |
| **Load** |  |  |  |  |  |  |
| 0KG - 105KG |  | 0.205 | 0.070 | 244.072 | 2.941 | 0.019 |
| 0KG - 130KG |  | 0.048 | 0.070 | 244.011 | 0.687 | 0.902 |
| 0KG - 80KG |  | 0.282 | 0.072 | 246.709 | 3.904 | 0.001 |
| 105KG - 130KG |  | -0.158 | 0.070 | 244.072 | -2.257 | 0.111 |
| 105KG - 80KG |  | 0.076 | 0.072 | 246.839 | 1.051 | 0.719 |
| 130KG - 80KG |  | 0.234 | 0.072 | 246.709 | 3.241 | 0.007 |
| **Total Proteins** |  |  |  |  |  |  |
| **Load** |  |  |  |  |  |  |
| 0KG - 105KG |  | -4.4 | 0.9 | 245.1 | -4.7 | 0.00003 |
| 0KG - 130KG |  | -2.5 | 0.9 | 245.0 | -2.7 | 0.04 |
| 0KG - 80KG |  | -1.9 | 1.0 | 247.4 | -2.0 | 0.20 |
| 105KG - 130KG |  | 1.9 | 0.9 | 245.1 | 2.1 | 0.17 |
| 105KG - 80KG |  | 2.5 | 1.0 | 247.5 | 2.6 | 0.05 |
| 130KG - 80KG |  | 0.6 | 1.0 | 247.4 | 0.6 | 0.93 |
| **GGT** |  |  |  |  |  |  |
| **Load** |  |  |  |  |  |  |
| 0KG - 105KG |  | -6.95 | 1.36 | 245.02 | -5.12 | <0.0001 |
| 0KG - 130KG |  | -9.36 | 1.35 | 245.00 | -6.93 | <0.0001 |
| 0KG - 80KG |  | 1.12 | 1.40 | 246.00 | 0.80 | 0.85 |
| 105KG - 130KG |  | -2.41 | 1.36 | 245.02 | -1.78 | 0.28 |
| 105KG - 80KG |  | 8.08 | 1.40 | 246.05 | 5.76 | <0.0001 |
| 130KG - 80KG |  | 10.49 | 1.40 | 246.00 | 7.51 | <0.0001 |
| **CK** |  |  |  |  |  |  |
| **Load** |  |  |  |  |  |  |
| 0KG - 105KG |  | -46.15 | 18.83 | 246.03 | -2.45 | 0.07 |
| 0KG - 130KG |  | -0.50 | 18.83 | 246.03 | -0.03 | 0.99 |
| 0KG - 80KG |  | -14.56 | 19.41 | 249.77 | -0.75 | 0.88 |
| 105KG - 130KG |  | 45.65 | 18.83 | 246.03 | 2.42 | 0.08 |
| 105KG - 80KG |  | 31.59 | 19.41 | 249.77 | 1.63 | 0.36 |
| 130KG - 80KG |  | -14.06 | 19.41 | 249.77 | -0.72 | 0.89 |
| **SAP** |  |  |  |  |  |  |
| **Load** |  |  |  |  |  |  |
| 0KG - 105KG |  | -25.53 | 6.24 | 245.06 | -4.09 | 0.0003 |
| 0KG - 130KG |  | -25.18 | 6.21 | 245.01 | -4.05 | 0.0004 |
| 0KG - 80KG |  | -21.12 | 6.41 | 247.46 | -3.29 | 0.006 |
| 105KG - 130KG |  | 0.35 | 6.24 | 245.06 | 0.06 | 0.99 |
| 105KG - 80KG |  | 4.41 | 6.44 | 247.57 | 0.68 | 0.90 |
| 130KG - 80KG |  | 4.06 | 6.41 | 247.46 | 0.63 | 0.92 |
| **LDH** |  |  |  |  |  |  |
| **Time*Load** |  |  |  |  |  |  |
| 0KG - 105KG | T1 | -28.3 | 75.5 | 244.0 | -0.4 | 0.98 |
| 0KG - 130KG | T1 | 23.1 | 75.5 | 244.0 | 0.3 | 0.99 |
| 0KG - 80KG | T1 | 2.9 | 77.3 | 244.5 | 0.0 | 1.00 |
| 105KG - 130KG | T1 | 51.3 | 75.5 | 244.0 | 0.7 | 0.90 |
| 105KG - 80KG | T1 | 31.2 | 77.3 | 244.5 | 0.4 | 0.98 |
| 130KG - 80KG | T1 | -20.1 | 77.3 | 244.5 | -0.3 | 0.99 |
| 0KG - 105KG | T2 | 112.9 | 77.3 | 244.4 | 1.5 | 0.46 |
| 0KG - 130KG | T2 | -6.7 | 75.5 | 244.0 | -0.1 | 1.00 |
| 0KG - 80KG | T2 | 30.1 | 77.3 | 244.5 | 0.4 | 0.98 |
| 105KG - 130KG | T2 | -119.6 | 77.3 | 244.4 | -1.5 | 0.41 |
| 105KG - 80KG | T2 | -82.8 | 79.0 | 244.9 | -1.0 | 0.72 |
| 130KG - 80KG | T2 | 36.7 | 77.3 | 244.5 | 0.5 | 0.96 |
| 0KG - 105KG | T3 | -54.7 | 75.5 | 244.0 | -0.7 | 0.89 |
| 0KG - 130KG | T3 | 58.1 | 77.3 | 244.5 | 0.8 | 0.88 |
| 0KG - 80KG | T3 | 5.7 | 77.3 | 244.5 | 0.1 | 1.00 |
| 105KG - 130KG | T3 | 112.8 | 77.3 | 244.5 | 1.5 | 0.46 |
| 105KG - 80KG | T3 | 60.5 | 77.3 | 244.5 | 0.8 | 0.86 |
| 130KG - 80KG | T3 | -52.4 | 78.9 | 244.0 | -0.7 | 0.91 |
| 0KG - 105KG | T4 | -204.0 | 75.5 | 244.0 | -2.7 | 0.03 |
| 0KG - 130KG | T4 | -24.0 | 75.5 | 244.0 | -0.3 | 0.99 |
| 0KG - 80KG | T4 | 46.9 | 77.3 | 244.5 | 0.6 | 0.93 |
| 105KG - 130KG | T4 | 180.0 | 75.5 | 244.0 | 2.4 | 0.08 |
| 105KG - 80KG | T4 | 250.9 | 77.3 | 244.5 | 3.2 | 0.007 |
| 130KG - 80KG | T4 | 70.9 | 77.3 | 244.5 | 0.9 | 0.80 |
| 0KG - 105KG | T5 | -331.3 | 75.5 | 244.0 | -4.4 | 0.0001 |
| 0KG - 130KG | T5 | -247.7 | 75.5 | 244.0 | -3.3 | 0.007 |
| 0KG - 80KG | T5 | 30.7 | 79.3 | 244.9 | 0.4 | 0.98 |
| 105KG - 130KG | T5 | 83.6 | 75.5 | 244.0 | 1.1 | 0.69 |
| 105KG - 80KG | T5 | 362.0 | 79.3 | 244.9 | 4.6 | <0.0001 |
| 130KG - 80KG | T5 | 278.4 | 79.3 | 244.9 | 3.5 | 0.002 |
| 0KG - 105KG | T6 | -366.0 | 75.5 | 244.0 | -4.8 | <0.0001 |
| 0KG - 130KG | T6 | -119.8 | 75.5 | 244.0 | -1.6 | 0.39 |
| 0KG - 80KG | T6 | -63.6 | 77.3 | 244.5 | -0.8 | 0.84 |
| 105KG - 130KG | T6 | 246.3 | 75.5 | 244.0 | 3.3 | 0.007 |
| 105KG - 80KG | T6 | 302.4 | 77.3 | 244.5 | 3.9 | 0.001 |
| 130KG - 80KG | T6 | 56.1 | 77.3 | 244.5 | 0.7 | 0.89 |
| **Load** |  |  |  |  |  |  |
| 0KG - 105KG |  | -145.22 | 30.96 | 244.08 | -4.69 | <0.0001 |
| 0KG - 130KG |  | -52.82 | 30.96 | 244.10 | -1.71 | 0.32 |
| 0KG - 80KG |  | 8.80 | 31.83 | 246.81 | 0.28 | 0.99 |
| 105KG - 130KG |  | 92.40 | 31.08 | 244.17 | 2.97 | 0.02 |
| 105KG - 80KG |  | 154.02 | 31.96 | 246.93 | 4.82 | <0.0001 |
| 130KG - 80KG |  | 61.62 | 31.90 | 246.00 | 1.93 | 0.22 |
| **GP** |  |  |  |  |  |  |
| **Load** |  |  |  |  |  |  |
| 0KG - 105KG |  | 38.310 | 6.263 | 228.117 | 6.117 | <0.0001 |
| 0KG - 130KG |  | 20.365 | 6.059 | 223.357 | 3.361 | 0.005 |
| 0KG - 80KG |  | 23.028 | 6.243 | 226.445 | 3.689 | 0.002 |
| 105KG - 130KG |  | -17.945 | 6.380 | 230.767 | -2.813 | 0.02 |
| 105KG - 80KG |  | -15.282 | 6.567 | 231.866 | -2.327 | 0.09 |
| 130KG - 80KG |  | 2.663 | 6.313 | 224.650 | 0.422 | 0.97 |
| **NL** |  |  |  |  |  |  |
| **Load** |  |  |  |  |  |  |
| 0KG - 105KG |  | -0.81 | 0.21 | 246.02 | -3.86 | 0.0008 |
| 0KG - 130KG |  | 0.26 | 0.21 | 246.11 | 1.24 | 0.60 |
| 0KG - 80KG |  | -0.12 | 0.22 | 248.96 | -0.56 | 0.94 |
| 105KG - 130KG |  | 1.08 | 0.21 | 246.11 | 5.08 | <0.0001 |
| 105KG - 80KG |  | 0.69 | 0.22 | 248.96 | 3.19 | 0.008 |
| 130KG - 80KG |  | -0.38 | 0.22 | 248.09 | -1.77 | 0.29 |
| **VGA** |  |  |  |  |  |  |
| **Load** |  |  |  |  |  |  |
| 0KG - 105KG |  | -0.35 | 0.55 | 246.01 | -0.65 | 0.92 |
| 0KG - 130KG |  | 0.48 | 0.55 | 246.08 | 0.88 | 0.82 |
| 0KG - 80KG |  | -1.14 | 0.56 | 248.35 | -2.02 | 0.18 |
| 105KG - 130KG |  | 0.84 | 0.55 | 246.08 | 1.52 | 0.43 |
| 105KG - 80KG |  | -0.78 | 0.56 | 248.35 | -1.39 | 0.51 |
| 130KG - 80KG |  | -1.62 | 0.56 | 247.65 | -2.87 | 0.02 |
| **Cortisol** |  |  |  |  |  |  |
| **Load** |  |  |  |  |  |  |
| 0KG - 105KG |  | -32.75 | 6.15 | 118.00 | -5.33 | <0.0001 |
| 0KG - 130KG |  | -34.38 | 6.15 | 118.00 | -5.59 | <0.0001 |
| 0KG - 80KG |  | -17.02 | 6.32 | 118.84 | -2.69 | 0.04 |
| 105KG - 130KG |  | -1.63 | 6.15 | 118.00 | -0.27 | 0.99 |
| 105KG - 80KG |  | 15.74 | 6.32 | 118.84 | 2.49 | 0.07 |
| 130KG - 80KG |  | 17.37 | 6.32 | 118.84 | 2.75 | 0.03 |
| **Glucose** |  |  |  |  |  |  |
| **Load** |  |  |  |  |  |  |
| 0KG - 105KG |  | -46.15 | 18.83 | 246.03 | -2.45 | 0.07 |
| 0KG - 130KG |  | -0.50 | 18.83 | 246.03 | -0.03 | 0.99 |
| 0KG - 80KG |  | -14.56 | 19.41 | 249.77 | -0.75 | 0.87 |
| 105KG - 130KG |  | 45.65 | 18.83 | 246.03 | 2.42 | 0.07 |
| 105KG - 80KG |  | 31.59 | 19.41 | 249.77 | 1.63 | 0.36 |
| 130KG - 80KG |  | -14.06 | 19.41 | 249.77 | -0.72 | 0.88 |
| **Lactate** |  |  |  |  |  |  |
| **Load** |  |  |  |  |  |  |
| 0KG - 105KG |  | -0.66 | 0.20 | 117.00 | -3.32 | 0.007 |
| 0KG - 130KG |  | -0.74 | 0.20 | 117.00 | -3.70 | 0.002 |
| 0KG - 80KG |  | -0.54 | 0.21 | 117.70 | -2.59 | 0.05 |
| 105KG - 130KG |  | -0.08 | 0.20 | 117.00 | -0.39 | 0.98 |
| 105KG - 80KG |  | 0.13 | 0.21 | 117.70 | 0.61 | 0.92 |
| 130KG - 80KG |  | 0.20 | 0.21 | 117.70 | 0.98 | 0.76 |
| **Temperature** |  |  |  |  |  |  |
| **Load** |  |  |  |  |  |  |
| 0KG - 105KG |  | -0.11 | 0.12 | 121.00 | -0.93 | 0.79 |
| 0KG - 130KG |  | -0.05 | 0.12 | 121.00 | -0.41 | 0.98 |
| 0KG - 80KG |  | -0.40 | 0.12 | 121.00 | -3.28 | 0.007 |
| 105KG - 130KG |  | 0.06 | 0.12 | 121.00 | 0.52 | 0.95 |
| 105KG - 80KG |  | -0.29 | 0.12 | 121.00 | -2.35 | 0.09 |
| 130KG - 80KG |  | -0.35 | 0.12 | 121.00 | -2.87 | 0.02 |
